# Hepatectomy After Conversion Therapy Using Tyrosine Kinase Inhibitors Plus Anti-PD-1 Antibody Therapy for Patients with Unresectable Hepatocellular Carcinoma

**DOI:** 10.1245/s10434-022-12530-z

**Published:** 2022-09-30

**Authors:** Xiao-Dong Zhu, Cheng Huang, Ying-Hao Shen, Bin Xu, Ning-Ling Ge, Yuan Ji, Xu-Dong Qu, Lingli Chen, Yi Chen, Mei-Ling Li, Jin-Jin Zhu, Zhao-You Tang, Jian Zhou, Jia Fan, Hui-Chuan Sun

**Affiliations:** 1 Department of Liver Surgery and Transplantation, Liver Cancer Institute and Zhongshan Hospital, Fudan University, Shanghai, China; 2 Department of Hepatic Oncology, Liver Cancer Institute and Zhongshan Hospital, Fudan University, Shanghai, China; 3 Department of Pathology, Zhongshan Hospital, Fudan University, Shanghai, China; 4 Department of Interventional Radiology, Zhongshan Hospital, Fudan University, Shanghai, China

## Abstract

**Background:**

Combined treatment with tyrosine kinase inhibitors (TKI) plus anti-PD-1 antibodies showed high anti-tumor efficacy and made conversion resection possible for patients with unresectable hepatocellular carcinoma (HCC). However, long-term survival has not been reported.

**Methods:**

A cohort of consecutive patients who received combined TKI/anti-PD-1 antibodies as first-line treatment for initially unresectable HCC at the authors’ hospital between August 2018 and September 2020 was eligible for this study. Patients who were responding to systemic therapy and met the criteria for hepatectomy underwent liver resection with curative intention. The study also investigated the association of clinical factors with successful conversion resection and postoperative recurrence.

**Results:**

The study enrolled 101 patients including 24 patients (23.8 %) who underwent R0 resection a median of 3.9 months (interquartile range: 2.5–5.9 months) after initiation of systemic therapy. Patients with an Eastern cooperative oncology group performance status of 0, fewer intrahepatic tumors, or a radiographic response to systemic therapy were more likely to be able to receive curative resection. After a median follow-up period of 21.5 months, hepatectomy was independently associated with a favorable overall survival (hazard ratio [HR], 0.050; 95 % confidence interval [CI], 0.007–0.365; *P* = 0.003). For the 24 patients who underwent surgery, the 12-month recurrence-free survival and overall survival rates were respectively 75% and 95.8%. Achieving a pathologic complete response (*n* = 10) to systemic therapy was associated with a favorable recurrence-free survival after resection, with a trend toward significance (HR, 0.345; 95% CI, 0.067–1.785; *P* = 0.187).

**Conclusions:**

Selected patients with initially unresectable HCC can undergo hepatectomy after systemic therapy with combined TKI/anti-PD-1 antibodies. In this study, conversion resection was associated with a favorable prognosis.

The incidence and mortality rates remain high for liver cancers, among which hepatocellular carcinoma (HCC) is the most common pathologic type. In China, most patients with a diagnosis of HCC have advanced-stage disease,^1^ which is the key driver behind the poorer long-term survival of Chinese HCC patients than their Japanese counterparts.^2^ Diagnosis at a late stage also precludes curative treatment for the majority of Chinese patients with HCC.

Recent years have seen the development of novel systemic therapies with high antitumor efficacy for unresectable or advanced HCC. Before September 2020, only two tyrosine kinase inhibitors (TKIs), sorafenib and lenvatinib, were approved for the first-line treatment of advanced HCC. However, the overall survival (OS) associated with these agents was approximately 1 year.^3,4^

More recently, combination therapy with an anti-angiogenic agent and an immune checkpoint inhibitor has shown potent antitumor efficacy, with objective response rates (ORRs) of 30% or higher. Among such combination therapies, lenvatinib or apatinib plus anti-programmed death-1 (PD-1) antibodies has shown ORRs of 36% and 34% respectively in phase 1b and 2 studies.^5,6^ Additionally, as we have demonstrated, real-world clinical practice shows that lenvatinib plus various anti-PD-1 antibodies has an ORR of 33.3% for patients with advanced or unresectable HCC.^7^

Thanks to these potent combination therapies for advanced HCC, tumor downsizing or even downstaging has become more common.^8–10^ This unprecedented high antitumor efficacy also has made conversion therapy possible for a proportion of patients with initially unresectable HCC at diagnosis.^11^ In a previous report, we presented 10 patients with initially unresectable HCC who were successfully converted to resectable disease and underwent safe curative resection.^9^ Data from the same study suggest that in real-world practice, first-line combined treatment with a TKI plus anti-PD-1 antibodies may enable about 20% of patients with initially unresectable HCC to undergo liver resection safely.^9^ However, no data on the long-term survival outcomes currently are associated with this conversion therapy strategy.

In the current study, we reviewed a large cohort of 101 patients with advanced/unresectable HCC who received first-line treatment with a TKI plus anti-PD-1 antibodies. This report describes the rate of patients who subsequently underwent R0 resection, the clinical features associated with clinical downstaging, and the long-term survival outcomes for the patients who underwent R0 resection.

## Materials and Methods

### Patients

In this observational study, data from consecutive patients with unresectable or advanced HCC who received first-line treatment with a TKI plus anti-PD-1 antibodies at our institution were collected into a prospectively established database. Unresectable disease was assessed by a multi-disciplinary treatment group and defined as having intermediate or advanced stage HCC (BCLC stage B or C) or as not able to tolerate curative liver resection (e.g., insufficient remnant liver volume after liver resection).

The study protocol complied with the ethical guidelines of the World Medical Association Declaration of Helsinki and was approved by Zhongshan Hospital Research Ethics Committee (Approval No. B2020-177R). All the patients provided written informed consent before receiving systemic therapy and before undergoing surgery.

### Treatment

Various oral TKIs were used in this study, including lenvatinib (8 mg/day regardless of body weight), apatinib (250 mg/day),^6^ and sorafenib (800 mg/day). The anti-PD-1 antibodies used in this study included pembrolizumab (200 mg q3w), nivolumab (3 mg/kg q2w), camrelizumab (200 mg q2w),^12^ toripalimab (240 mg q3w), and sintilimab (200 mg q3w).^13^ Because the preliminary efficacy and safety data for the lenvatinib/pembrolizumab combination was encouraging,^14^ lenvatinib was the preferred TKI, and the type of anti-PD-1 antibody used was based on the patient’s choice after a full discussion on the latest efficacy and safety data, treatment cycle, and cost.

To manage adverse events, dose modification or discontinuation was permitted. For patients with untreated hepatitis B virus (HBV) infection, an oral anti-viral agent (entecavir or tenofovir) was given and continued throughout anti-cancer treatment. Systemic treatment was continued until tumor progression, development of unacceptable toxicity, or interruption when curative liver resection was received.

### Clinical Assessments

Tumor assessments were performed every 2 months (range, 8–9 weeks). Intrahepatic tumors and upper abdominal metastasis were assessed with contrast-enhanced magnetic resonance imaging (MRI) or computed tomography (CT), and lung metastases were assessed with chest CT. Response Evaluation Criteria in Solid Tumours (RECIST) v1.1 and the HCC-specific modified RECIST (mRECIST) were used for tumor assessment.

### Indication and Procedures of Liver Resection

Liver resection was indicated according to the following uniform criteria. (1) Intrahepatic tumors were evaluated as partial response (PR) or stable disease (SD) for at least 2 months. (2) Extrahepatic lesions and vascular embolus were technically resectable. (3) An R0 resection could be achieved with sufficient remnant liver volume (≥ 40 % of standard liver volume for patients with liver cirrhosis or ≥ 30 % of standard liver volume for patients without liver cirrhosis).^15^ (4) No severe or persistent adverse events occurred from TKI or anti-PD-1 antibody. (5) The patient had no contraindications for hepatectomy.

For the patients who planned to undergo liver resection, TKIs were discontinued for at least 1 week, and anti-PD-1 antibodies were discontinued for at least 3 weeks. A pre-surgery liver biopsy of non-tumoral liver tissue was mandatory to exclude underlying liver inflammation. A pathologic complete response (pCR) was defined as no residual viable tumor cells with hematoxylin and eosin staining on slide sections from completely resected primary tumors, tumor thrombosis, or metastatic lesions.

Both TKIs and anti-PD-1 antibodies were resumed approximately 4 weeks after hepatectomy when patients were fully recovered from surgery. Postoperative radiographic assessment was performed every 2 months in the first year and every 3–4 months in the following year. If no evidence of tumor recurrence was found, the combination therapy was discontinued 8–10 months after surgery, following discussion with the patient.

When tumor recurrence was diagnosed, the choice of treatment after recurrence was based on the pattern of tumor recurrence.

### Statistical Analysis

Differences in categorical variables were evaluated for statistical significance using the chi-square test or Fisher’s exact test, where appropriate. Continuous variables were expressed as the median (interquartile range [IQR]) or as the mean (standard deviation) and compared using Student’s *t* test. Overall survival was defined as the interval between the date of initiation of combination therapy and the patient’s death. Time-to-recurrence was defined as the interval between the date of surgery and the date of the diagnosis of tumor recurrence. If the patient died of causes other than liver disease, the patient was censored at the date of the patient’s death.

The Kaplan–Meier method was used to estimate the survival rates at each time point. The log-rank test was used to compare OS and time-to-recurrence between groups. A Cox regression model was used to perform multivariate analysis. A *P* value lower than 0.05 was considered statistically significant. Statistical analyses were performed using PASW Statistics v.18.0 for Windows (IBM Corp., Armonk, NY, USA) or R v3.1.0 (http://www.r-project.org).

## Results

### Patient Characteristics at Baseline

The study enrolled 101 consecutive eligible patients with unresectable or advanced HCC who received first-line systemic therapy with combined TKI plus anti-PD-1 antibodies between 15 August 2018 and 1 September 2020 (Table 1). The patients had a mean age of 54.8 ± 10.3 years. The majority were male (90.1%), and the etiology for most of the patients (96%) was HBV infection (serum HBsAg, HBcAb, or HBeAb positivity). Most of the patients (93.1%) were categorized as Child-Pugh class A. Among the study cohort, 37.6% of the patients had an Eastern Cooperative Oncology Group (ECOG) performance status (PS) score of 0, 23.8% and 72.3% had a diagnosis of Barcelona Clinic Liver Cancer (BCLC) stage B and C HCC. Macroscopic vascular invasion (MVI) occurred for 56.4% of the patients and extrahepatic spread (EHS) for 28.7% of the patients. Most of the patients received lenvatinib (85.1%), with 12.9% receiving apatinib and 2% receiving sorafenib.

**Table 1:** Baseline patient demographics and disease characteristics

Characteristic	Total (*n* = 101) *n* (%)	Patients who underwent hepatectomy (*n* = 24) *n* (%)	Patients who did not undergo hepatectomy (*n* = 77) *n* (%)	*P* value
Sex (male/female)	91 (90.1)/10 (9.9)	22 (91.7)/2 (8.3)	69 (89.6)/8 (10.4)	1.000
Mean age (standard deviation) (years)	54.8 (10.3)	52.9 (6.8)	55.4 (11.1)	0.182
ECOG performance status (0/≥1)	38 (37.6)/63 (62.4)	14 (58.3)/10 (41.7)	24 (31.2)/53 (68.8)	0.016
Etiology of HCC (HBV/HCV/non-viral)	97 (96.0 %)/0 (0.0)/4 (4.0)	23 (95.8)/0 (0.0)/1 (4.2)	74 (96.1)/0 (0.0)/3 (3.9)	1.000
BCLC stage (A/B/C)	4 (4.0)/24 (23.8)/73 (72.3)	3 (12.5)/6 (25.0)/15 (62.5)	1 (1.3)/18 (23.4)/58 (75.3)	0.052
China liver cancer stage (1b/2a/2b/3a/3b)	4 (4.0)/4 (4.0)/20 (19.8)/44 (43.6)/29 (28.7)	3 (12.5)/1 (4.2)/5 (20.8)/11 (45.8)/4 (16.7)	1 (1.3)/3 (3.9)/15 (19.5)/33 (42.9)/25 (32.5)	0.027^a^
Macrovascular invasion (yes/no)	57 (56.4)/44 (43.6)	13 (54.2)/11 (45.8)	44 (57.1)/33 (42.9)	0.797
Extrahepatic disease (yes/no)	29 (28.7)/72 (71.3 %)	4 (16.7)/20 (83.3)	25 (32.5)/52 (67.5)	0.135
Child-Pugh class (A/B)	94 (93.1)/7 (6.9)	23 (95.8)/1 (4.2)	71 (92.2)/6 (7.8)	0.683
Baseline AFP ≥400 ng/mL	60 (59.4)	17 (70.8)	43 (55.8)	0.192
Baseline PIVKA-II ≥1000 mAU/mL	71 (70.3)	17 (70.8)	54 (70.1)	0.947
Intrahepatic tumor number (1/2–3/≥4)	22 (21.8)/30 (29.7)/49 (48.5)	10 (41.7)/6 (25.0)/8 (33.3)	12 (15.6)/24 (31.2)/41 (53.2)	0.024
Objective response, per RECIST v1.1 criteria (yes/no or not evaluable)	33 (32.7)/68 (67.3)	12 (50.0)/12 (50.0)	21 (27.3)/56 (72.7)	0.038
Objective response, per mRECIST criteria (yes/no or not evaluable)	50 (49.5)/51 (50.5 %)	18 (75.0)/6 (25.0)	32 (41.6)/45 (58.4)	0.004

*ECOG* eastern cooperative oncology group, *HCC* hepatocellular carcinoma, *HBV* hepatitis B virus, *HCV* hepatitis C virus, *BCLC* Barcelona clinic liver cancer, *AFP* alpha-fetoprotein, *PIVKA-II* protein induced by vitamin K absence-II, *RECIST* response evaluation criteria in solid tumors, *mRECIST* modified RECIST

^a^Linear-by-linear association

### Tumor Response and Patient Survival

At the data cutoff on 30 May 2022, the median follow-up time was 21.5 months (IQR, 7.8–28.8 months). The ORR was 32.7% (33/101; 2 complete responses, 31 partial responses, 47 cases of stable disease, 15 cases of progressive disease, and 6 patients without any tumor assessment) according to RECIST v1.1 or 49.5% (50/101; 10 complete responses, 40 partial responses, 30 cases of stable disease, 15 cases of progressive disease, and 6 patients without any tumor evaluation) according to mRECIST.

**Fig. 1:**
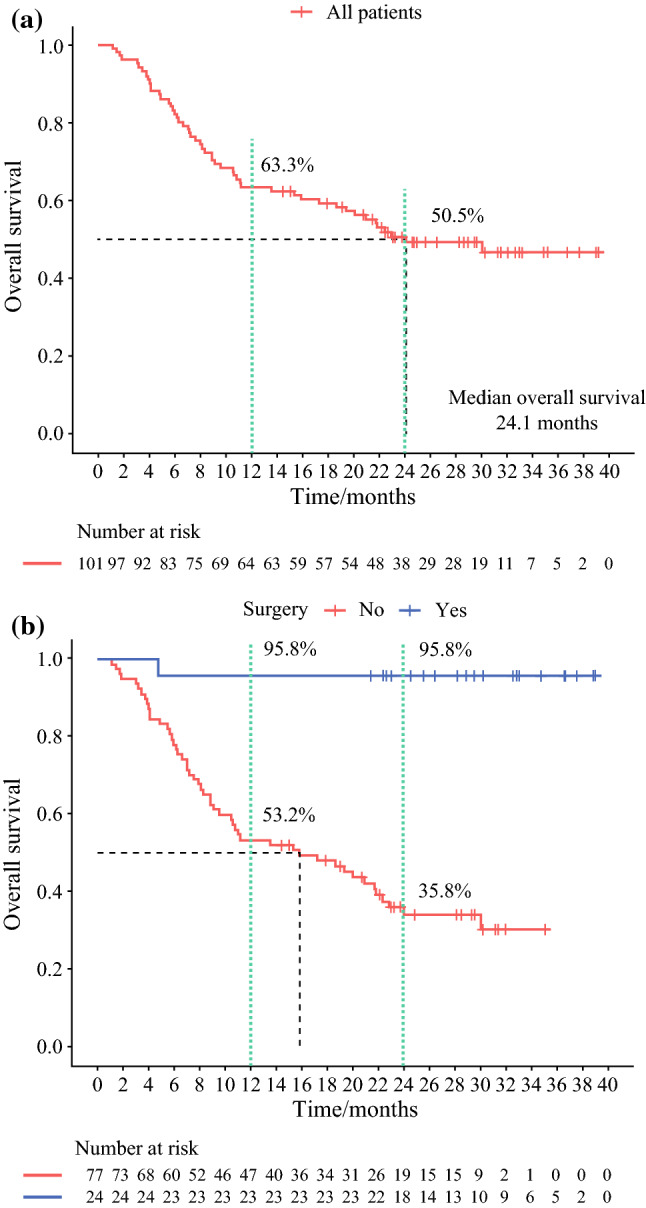
Cumulative survival plots after systemic therapy initiation for **a** all patients (*n* = 101) and **b** patients who underwent (*n* = 24) or did not undergo (*n* = 77) conversion surgery.

**Fig. 2:**
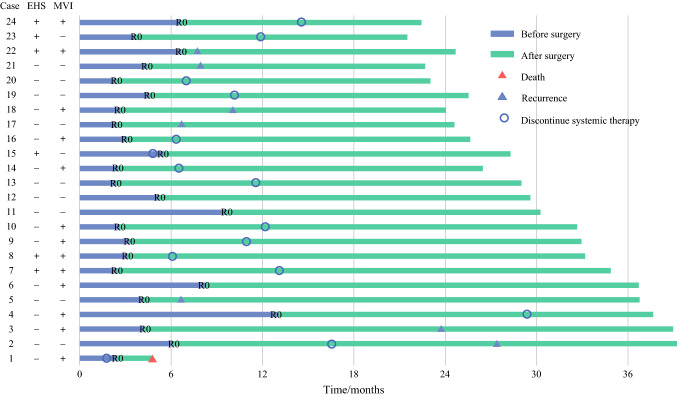
Swimmer plots of the 24 patients who underwent conversion resection. EHS, extrahepatic spread; MVI, macrovascular invasion; R0, R0 resection

At the data cutoff, 51 patients had died, and the estimated median OS for all 101 patients was 24.1 months (Fig. 1a). During a median time of 3.9 months (IQR, 2.5–5.9 months) after the initiation of systemic therapy, 24 patients (23.8%) underwent curative resection (Fig. 2). The patients who underwent resection had longer OS than those who did not (median OS not reached vs 15.9 months [95% CI, 7.0–24.7 months]; *P* < 0.001; Fig. 1b).

In the multivariate analysis, resection was independently associated with a favorable OS (hazard ratio [HR], 0.050; 95% confidence interval [CI], 0.007–0.365; *P* = 0.003) and a favorable tumor response as assessed by RECIST v1.1 criteria (*P* = 0.004) or mRECIST criteria (*P* = 0.001) as well as with macrovascular invasion (*P* = 0.050) and ECOG PS (*P* = 0.019) (Table 2).

**Table 2:** The associations between clinical factors and patient survival

Characteristics	Univariate analysis	Multivariate analysis		
	*P* Value	HR	95 % CI	*P* value
Sex (male vs female)	0.204			
Age (≥60 vs <60 years)	0.256			
ECOG performance status (0 vs 1–2)	< 0.001	0.421	0.204–0.869	0.019
Etiology of HCC, HBV vs HCV or non-viral	0.770			
BCLC stage (A vs B vs C)	0.108			
Macrovascular invasion (yes vs no)	0.044	1.846	0.999–3.414	0.050
Extrahepatic disease (yes vs no)	0.445			
Child-Pugh class (A vs B)	0.082			
Baseline AFP (≥400 vs <400 ng/mL)	0.267			
Baseline PIVKA-II (≥1000 vs<1000 mAU/mL)	0.272			
Intrahepatic tumor number (1–3 vs ≥4)	0.030	0.676	0.386–1.183	0.170
Objective response per RECIST v1.1 (response vs non-response or not evaluable)	0.005	0.360	0.180–0.723	0.004
Objective response per mRECIST criteria (response vs non-response or not evaluable)	< 0.001	0.367	0.201–0.670	0.001
Surgical resection (yes vs no)	< 0.001	0.050	0.007–0.365	0.003

*HR* hazard ratio, *CI* confidence interval, *ECOG* eastern cooperative oncology group, *HCC* hepatocellular carcinoma, *HBV* hepatitis B virus, *HCV* hepatitis C virus, *BCLC* Barcelona clinic liver cancer, *AFP* alpha-fetoprotein, *PIVKA-II* protein induced by vitamin K absence-II, *RECIST* response evaluation criteria in solid tumours, *mRECIST* modified RECIST

### Outcomes of Patients Who Underwent Hepatectomy

Of the 24 patients who underwent hepatectomy, 15 (62.5%) received an initial diagnosis of BCLC stage C disease, 13 (54.2%) had vascular invasion, and 4 (16.7%) had extrahepatic metastasis (Table 1). Generally, surgical resection was safe.^16^ All the patients recovered well from resection except for one patient who died of multi-system immune-related adverse effects 2.4 months after surgery, as previously reported.^9^ According to the pathologic study, 10 (41.7%) of the patients achieved a pCR after systemic therapy.

The baseline characteristics of the patients who received hepatectomy were compared with those of the patients who did not (Table 1). The patients who achieved an objective tumor response to systemic therapy per RECIST v1.1 criteria had a higher likelihood of undergoing a hepatectomy than those without a tumor response or those who were not evaluable (36.4% vs 17.6%; *P* = 0.038). The patients with a tumor response per mRECIST criteria also had a higher likelihood of undergoing hepatectomy than those without a response (36.0% vs 11.8%; *P* = 0.004).

Other factors, including better ECOG PS (*P* = 0.016), earlier China liver cancer stage (*P* = 0.027), and fewer intrahepatic tumor nodules (*P* = 0.024), also were associated with a higher likelihood of undergoing hepatectomy. In the logistic regression analysis, a PS of 0 (odds ratio [OR], 2.814; 95% CI, 1.055–7.504; *P* = 0.039) and tumor response per mRECIST criteria (OR, 3.918; 95% CI, 1.372–11.194; *P* = 0.011) were independently associated with a high likelihood of hepatectomy.

After resection, 22 patients restarted systemic therapies, and 2 patients did not because of immune-related adverse effects (*n* = 1) or because of the patient’s own choice (*n* = 1) (Fig. 2). At the data cutoff, 14 (60.9%) of 23 living patients had discontinued systemic therapies, and 13 patients remained disease free (Fig. 2).

Of the 24 patients who underwent liver resection, the median postoperative follow-up time was 23.4 months (IQR, 20.7–30.0 months). One patient (case 1) had died of multi-system immune-related adverse effects at the time of data cutoff, whereas all the remaining patients were alive. The median OS after surgery or after the initiation of systemic therapy was not reached. The 12- and 24-month survival rates after surgery were respectively 95.8% and 95.8% (Fig. 1b).

**Fig. 3:**
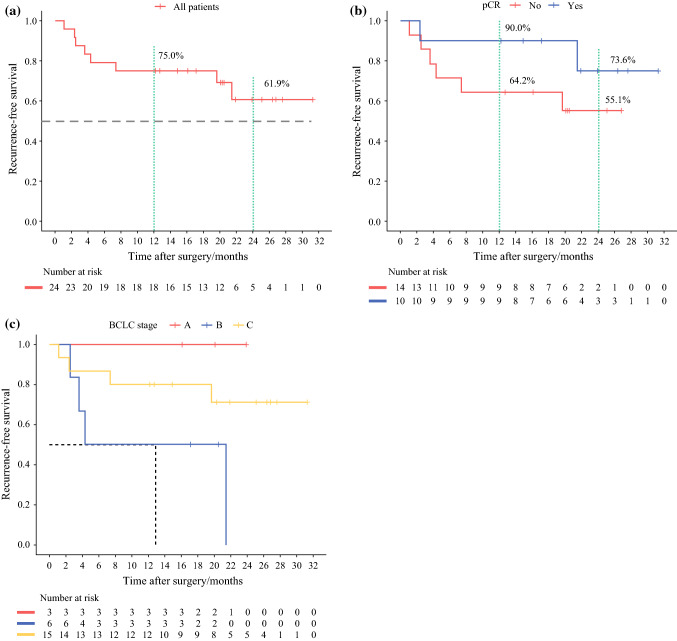
**a** Cumulative plot of recurrence-free survival for patients who underwent conversion resection. **b** Patients with a pathologic complete response (pCR) had a trend toward a favorable recurrence-free survival versus those without pCR. **c** The BCLC stages at baseline were not associated with recurrence-free survival.

Tumor recurrence was diagnosed for seven patients after resection (6 intrahepatic recurrences and 1 retroperitoneal lymph node metastasis). The median recurrence-free survival was not reached. The 12- and 24-month recurrence-free survival rates were respectively 75% and 61.9% (Fig. 3a). Compared with the patients without a pCR (*n* = 14), the patients who achieved a pCR (*n* = 10) had a trend toward a favorable recurrence-free survival (HR, 0.345; 95% CI, 0.067–1.785; *P* = 0.187; Fig. 3b). The 1- and 2-year recurrence-free survival rates were respectively 90.0% and 73.6% for the patients with pCR versus 64.2% and 55.1% for the patients without a pCR (Fig. 3b). Among the patients who achieved a pCR, only one recurrence was diagnosed (retroperitoneal lymph node metastasis) 21.4 months after resection or 10.8 months after the end of postoperative systemic therapy (case 2) (Fig. 2). Other factors, including the BCLC stage at baseline, were not associated with time to recurrence (*P* = 0.092; Fig. 3c).

## Discussion

The current study found that 23.8% of the patients with initially unresectable or advanced HCC were converted to resectable disease after combination therapy with a TKI and anti-PD-1 antibodies. The follow-up data for the patients who underwent successful hepatectomy after conversion therapy demonstrated that long-term and disease-free survival without any antitumor treatment could be achieved for patients with a diagnosis of advanced-stage HCC.

The current study supports the feasibility and potential benefit of a more aggressive treatment approach (e.g., systemic therapy followed by resection when tumors have responded to systemic therapy in patients with initially unresectable or advanced HCC). In the current study, the median survival time for all 101 patients was 24.1 months, which is numerically higher than the 19.2 months reported with atezolizumab plus bevacizumab in the IMbrave150 trial^17^ and 22.4 months reported with lenvatinib plus pembrolizumab in the KEYNOTE-524 trial.^5^ This difference in survival times may have been due to the fact that more than 20% of the patients in the current study underwent R0 resection, which is much higher than in the IMbrave150 or KEYNOTE-524 trials. For example, 1.8% of the patients received curative therapies after atezolizumab and bevacizumab in the IMbrave150 study, including 0.3% who received radiofrequency ablation and 1.5% who received resection.^18^ Furthermore, the median durations of response to bevacizumab/atezolizumab and lenvatinib/pembrolizumab in IMbrave150 and KEYNOTE-524 were 18 and 12 months, respectively.^5,18^ These durations are shorter than the median recurrence-free survival periods of the patients in the current study who underwent hepatectomy (> 24 months; Fig. 3a), suggesting that curative resection may provide a longer duration of tumor response.

Finally, 14 of the 24 patients in the current study who underwent resection had stopped systemic therapy at the data cutoff, suggesting that systemic therapy followed by curative resection can provide a long-term “cancer-free and drug-free survival.”^19^ Our findings align with a report by Kudo^19^ demonstrating potentially curative outcomes for patients with intermediate-stage HCC receiving systemic therapy with lenvatinib or atezolizumab/bevacizumab followed by curative transarterial chemoembolization. However, the current study suggests that curative outcomes are possible also for patients with advanced stage HCC (62.5% of the patients in our study had BCLC stage C disease). In the BCLC 2022 update, the strategy of “treatment stage migration” was proposed, which refers to patients with treatment failure who are migrated to the treatment recommendations for a more advanced disease stage even if the tumor stage has not changed.^20^ Because novel potent systemic therapies have recently been introduced for advanced or unresectable HCC, a reverse “treatment stage migration” may be possible; patients with advanced-stage HCC who show a strong response to systemic therapy may be able to receive curative therapies otherwise recommended only for early-stage HCC.

The findings of this study alone may not be sufficient to support the role of resection in prolonging survival after tumor downsizing or downstaging via systemic therapy. Indeed, the high postoperative survival rate observed in the current study could mainly have been a result of preoperative systemic therapy because most of the patients who underwent resection already had a deep tumor response (50% per RECIST v1.1 or 75% per mRECIST). According to the updated results from the IMbrave150^21^ and KEYNOTE-524 trials,^22^ patients with a confirmed response per RECIST 1.1 achieved long-term survival, in many cases longer than 24 months. Furthermore, a pCR, meaning no viable tumor cells were found in surgical specimens, was observed for 10 of the 24 patients in the current study who underwent liver resection, suggesting that HCC can be cured by systemic therapy alone. Therefore, the value of sequential resection needs to be investigated across patient groups, such as patients with pCR versus those without pCR. Nevertheless, the 2-year survival rate for the patients who had a response to the lenvatinib/pembrolizumab combination in the KEYNOTE-524 trial was less than 80%.^22^ In contrast, the 2-year survival rate after the initiation of systemic therapy for the 24 patients who underwent hepatectomy in the current study was 95.8% (Fig. 1b), although half of these patients were evaluated as non-responders to systemic therapy before surgery.

To address this question, we have initiated a randomized clinical trial to evaluate the effectiveness of surgical resection after systemic therapy with atezolizumab plus bevacizumab,^23^ which not only will provide evidence to support or dismiss the value of hepatectomy in this scenario, but also may identify which patients are most likely to benefit from sequential hepatectomy.

The current study also provided data on neoadjuvant therapy, which usually refers to preoperative systemic therapy for patients with technically resectable HCC. We found that the patients who achieved a pCR to systemic therapy had a longer time to recurrence than those without a pCR. This finding accords with results from Ho et al.^10^ who reported that a pathologic response after cabozantinib/nivolumab combination therapy for patients with locally advanced HCC was associated with prolonged progression-free survival. The aim of neoadjuvant therapy is to achieve a deep pathologic response to lower the recurrence rate after surgery. Our previous study demonstrated that the depth of radiographic tumor response was associated with the pathologic response of the patients treated with lenvatinib plus anti-PD-1 antibody therapy.^24^ For patients showing a tumor regression after systemic therapy, adequate treatment may be necessary to achieve a deep radiographic tumor response and, therefore, a deep pathologic response.

This study had several limitations. First, many of the combinations of TKIs and anti-PD-1 antibodies used in this study are not considered a standard of care for the first-line treatment of unresectable HCC. However, atezolizumab plus bevacizumab^18^ is approved for this indication in most countries, and sintilimab plus bevacizumab^13^ is approved in China. Furthermore, early-phase non-randomized clinical studies have shown high antitumor efficacy with most of the combinations used in this study. In addition, phase 3 studies evaluating the effectiveness of the combination of lenvatinib or apatinib with anti-PD-1 antibodies (NCT03713593 and NCT03764293) are ongoing. We currently are accumulating experience with downstaging using the bevacizumab-based combination therapy (NCT04843943, NCT04649489).

Second, most of the patients in this study had HBV-related HCC, which is characterized by relatively mild liver cirrhosis compared with HCV-related HCC. Patients with HBV-related HCC may therefore benefit more from immune-based therapies than patients with non-alcoholic steatohepatitis-related HCC.^25^ For this reason, evidence for conversion therapy for patients with HBV-related HCC may not be generalized to HCC with other etiologies.

Finally, some emerging data are showing the value of locoregional therapies (e.g., transarterial chemoembolization or radiotherapy) in downstaging or downsizing HCC tumors,^26–28^ and locoregional therapies may play an important role in conversion therapy, either alone or in combination with systemic therapies. However, the current study did not include patients who had received locoregional therapy.

In conclusion, together with the study by Shindoh et al.,^29^ the current study showed that hepatectomy after systemic therapy is feasible for patients with unresectable or advanced HCC, and preliminary follow-up data support the possibility of long-term cancer-free and drug-free survival with this treatment method.
